# The industrial feasibility of low temperature DeNO_*x*_ in the presence of SO_*x*_: a project case in a medium coking plant

**DOI:** 10.1039/c8ra02767f

**Published:** 2018-05-18

**Authors:** Changming Li, Jian Yu, Yi He, Chao Yu, Ping Li, Chao Wang, Fulin Huang, Shiqiu Gao

**Affiliations:** State Key Laboratory of Multi-phase Complex Systems, Institute of Process Engineering, Chinese Academy of Sciences Beijing 100190 China yujian@ipe.ac.cn +86-10-82544886; Solid Waste and Chemicals Management Center, Ministry of Environmental Protection of the People’s Republic of China Beijing 100029 China; School of Chemical Engineering, Xiangtan University Xiangtan Hunan 411105 China

## Abstract

Catalyst poisoning by SO_*x*_ has hindered the industrial application of selective catalytic reduction (SCR) technology for the DeNO_*x*_ of low temperature flue gas for many decades. The current engineering process of placing the SCR unit after the desulphurization and dedusting units can lead to high project and operational costs owing to low DeNO_*x*_ efficiency at low temperatures. Based on our previous pilot results, a DeNO_*x*_ project case was built before the desulfurization unit in a medium coking plant to explore the industrial feasibility of low temperature DeNO_*x*_ in the presence of SO_*x*_. A new engineering process was considered and designed to overcome the problem of SO_*x*_, including the elimination of SO_3_ with NH_3_ before the SCR reactor, the filtration of ash by a foam metal plate and *in situ* regeneration technology. The project case could run continuously for six months with above 70% DeNO_*x*_ efficiency and less than 10 ppm NH_3_ slip at 250 °C and a space velocity of 4000 h^−1^ in the presence of 260–300 mg m^−3^ SO_*x*_. The activity loss for the catalyst itself was not obvious after it had been running for six months, but blocking of the honeycomb channels by the sedimentary ash on the honeycomb catalyst modules owing to the low linear velocity resulted in decreased DeNO_*x*_ efficiency and an increased pressure drop. A improved DeNO_*x*_ process with gravitational dust collectors was also proposed to upgrade the present DeNO_*x*_ project case for further continuous and stable operation.

## Abstract 2

A DeNO_*x*_ project case was built to explore the industrial feasibility of low temperature DeNO_*x*_ in the presence of SO_*x*_.

## Graphical abstract

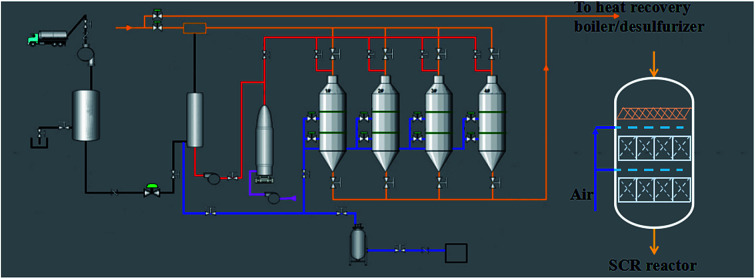

## 1. Introduction

The DeNO_*x*_ of low temperature flue gas from medium/small boilers is an urgent task in China because of increasingly strict NO_*x*_ emission regulations in recent years.^1–4^ Unlike the well-developed engineering application of selective catalytic reduction (SCR) technology for high temperature flue gas from power plants, great obstacles still restrict its wide industrial application in medium/small boilers because of the unsatisfactory catalytic activity and stability resulting from the unavoidable formation of ammonium sulfate species in the presence of SO_*x*_ at low temperatures.^5–8^ The flue gas has to undergo desulphurization and dedusting before the SCR units to avoid sulfur poisoning of the SCR catalyst, but the temperature of the flue gas may fall from more than 250 °C to less than 200 °C.^9,10^ The low temperature flue gas has to be reheated, as well as more catalyst used, with low space velocity for sufficient DeNO_*x*_ efficiency, which significantly increases the project and operation costs. As far as we know, however, all of the established SCR units are placed after the desulphurization and dedusting units for the purification of low temperature flue gas from medium/small boilers in China.

Our previous results from a SCR pilot test in a coking plant revealed that the deactivation of the honeycomb catalyst was mainly caused by severe coverage of the catalyst surface with ferric sulfate and ammonium sulfate species in the presence of SO_*x*_ below 270 °C.^11^ The ferric sulfate was from the corrosion of iron pipe by SO_3_ and ammonium sulfate was the product of a reaction between the injected NH_3_ and SO_3_ (but not SO_2_). But after SO_3_ and the ash with these sulfate species were eliminated using a dry desulfurization–dedusting process, the catalyst could even work with stable activity at 220 °C in the presence of high-concentration SO_2_. This previous work signifies that low temperature DeNO_*x*_ in the presence of SO_*x*_ may be industrially feasible if the problems of SO_3_ removal and pipeline corrosion are resolved by new engineering technology.

In this work, a DeNO_*x*_ project case was built before the desulfurization unit in a medium coking plant. A long flue of more than 50 meters was reserved between the locations of the injecting NH_3_ and SCR reactors, in which SO_3_ could be eliminated by the injected NH_3_ to form ammonium sulfate species through the gas-phase reaction between SO_3_ and NH_3_ with enough residence time. All the flues underwent antiseptic treatment using heat resistant paint. A replaceable foam metal plate was placed above the honeycomb catalyst in the SCR reactors to trap the ash and homogenize the flue gas. Moreover, an *in situ* regeneration process was also considered and designed to regenerate the deactivated catalyst with hot gas. In this way, this DeNO_*x*_ project case is expected to overcome the adverse effects of sulfate covering and has attractive technological advantages such as higher DeNO_*x*_ efficiency, lower amounts of catalyst used, and lower operation costs.

## 2. Technological process of the project case

**Fig. 1:**
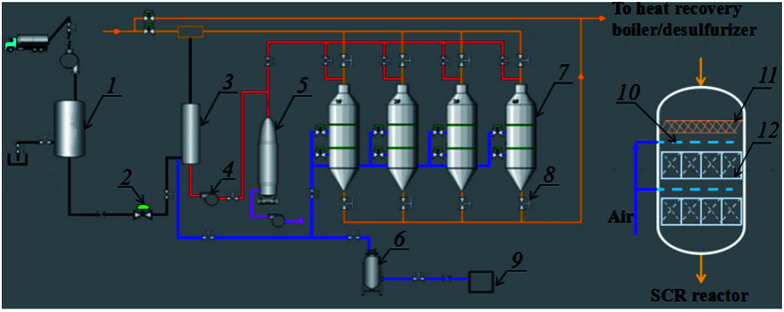
The technological process of the DeNO_*x*_ project case together with the details of a single SCR reactor: (1) ammonia tank, (2) motorized valve, (3) ammonia evaporator, (4) draught fan, (5) hot-blast stove, (6) air reservoir, (7) SCR reactor, (8) hand valve, (9) air compressor, (10) soot blower, (11) foam metal plate and (12) honeycomb catalyst.

In the project case, the SCR unit was placed before the desulphurization and dedusting units; the detailed technological process of the SCR unit is shown in Fig. 1. The SCR unit mainly included an aqua ammonia system, a heating system, a compressed air system and SCR reactors. 20 wt% aqua ammonia was used to provide NH_3_ for the SCR reaction. The aqua ammonia must be sufficiently gasified at a temperature above 200 °C in an ammonia evaporator before it can be injected into the main pipe, because unevaporated water droplets have been confirmed to facilitate the rapid formation of ammonium sulfates, in a similar way to the ammonia desulfurization process taking place through the main reactions in eqn (1) and (2).^12^ Moreover, the pipe length between the entrance for injecting NH_3_ and the SCR reactors was more than 50 meters, which provided enough residence time for the gas-phase reaction between NH_3_ and a small amount of SO_3_ through the reaction in eqn (3). This design could eliminate SO_3_ using NH_3_ before the flue gas was introduced into the SCR reactors and avoid the direct formation of ammonium sulfate species on the catalytic surface between the adsorbed ammonia and gaseous SO_3_. The produced ammonium sulfate dust would be trapped by the replaceable foam metal plate placed above the honeycomb catalyst in SCR reactors.

1 2NH_3_ + H_2_O + SO_2_ → (NH_4_)_2_SO_3_

2 (NH_4_)_2_SO_3_ + 0.5O_2_ → (NH_4_)_2_SO_4_

3 2NH_3_ + H_2_O + SO_3_ → (NH_4_)_2_SO_4_

The high-temperature gas (>450 °C) from the hot-blast stove was used to heat and vaporise aqua ammonia, or *in situ* regenerate the honeycomb catalyst in the SCR reactors through the decomposition of the ammonium sulfate species above 450 °C.^13,14^ Compressed air was supplied for the ammonia evaporator to dilute the vaporized NH_3_ and for the soot blower to clean the ash deposited on the catalytic surface. Four SCR reactors were designed for use alternately on a line. Two of the SCR reactors were kept spare under normal operating conditions, and the spare reactors would start being used when the catalyst in the used reactors needed to be regenerated by being heated. Thus, the SCR unit can operate continuously even if the stability of the catalyst is retained for only several months. In addition, a replaceable foam metal plate was placed above the honeycomb catalyst in each SCR reactor, which was used to trap the ammonium sulfate species and other dust to avoid coverage and blocking of the honeycomb catalyst. In this way, the design of the project case is expected to overcome the disadvantage of catalyst deactivation resulting from sulfur poisoning at low temperatures.

In the project case, a V based honeycomb catalyst was used, and the catalyst composition, as well as information on the specific surface and pore structure, is provided in Table 1. The V based catalyst was chosen by considering that the used catalyst poisoned by ammonium sulfate species can easily undergo thermal regeneration below 450 °C, which could be realized in the *in situ* regeneration process in the project case.

**Table 1:** The physicochemical properties of the produced honeycomb catalyst

Catalyst	Composition (wt%)^a^					BET data		
	V_2_O_5_	WO_3_	TiO_2_	SO_3_	Fe_2_O_3_	Specific area (m^2^ g^−1^)	Pore size (nm)	Pore volume (cm^3^ g^−1^)
Value	2.3	1.9	78.3	1.1	0.1	41.9	20.3	0.2

a Determined using XRF.

The parameters of the flue gas and designed craft in the DeNO_*x*_ project case are listed in Table 2. The concentration of NO_*x*_ in the original flue gas was in the range 400–500 mg m^−3^, and it needed to decrease to below 150 mg m^−3^ through the SCR reactors. The operation temperature of the DeNO_*x*_ reactor was in the range 240–270 °C, normally about 250 °C. The flue gas also contained 200–300 mg m^−3^ SO_2_ and a small amount of SO_3_ (below 5 ppm). In accordance with previous results from our pilot test,^11^ the gaseous hourly space velocity (GHSV) was designed to be 4000 h^−1^, and the desired DeNO_*x*_ efficiency would have been more than 70% with less than 150 mg m^−3^ NO_*x*_ in the exit of the SCR reactors. The flow of ammonia was tuned according to the NH_3_ slip in the exit of the SCR reactors, which was controlled below 10 ppm.

**Table 2:** The parameters of the flue gas and designed craft in the DeNO_*x*_ project case

Parameters	Composition of the flue gas					Temperature	Dust content	Flow	GHSV	DeNO_*x*_ efficiency
	NO_*x*_	SO_2_	SO_3_	H_2_O	O_2_					
Value	400–500 mg Nm^−3^	200–300 mg Nm^−3^	5–10 mg Nm^−3^	10–20 vol%	10–13 vol%	240–270 °C	∼30 mg Nm^−3^	∼180 000 Nm^3^	4000 h^−1^	>70%

## 3. Operating data for the DeNO_*x*_ project case

**Fig. 2:**
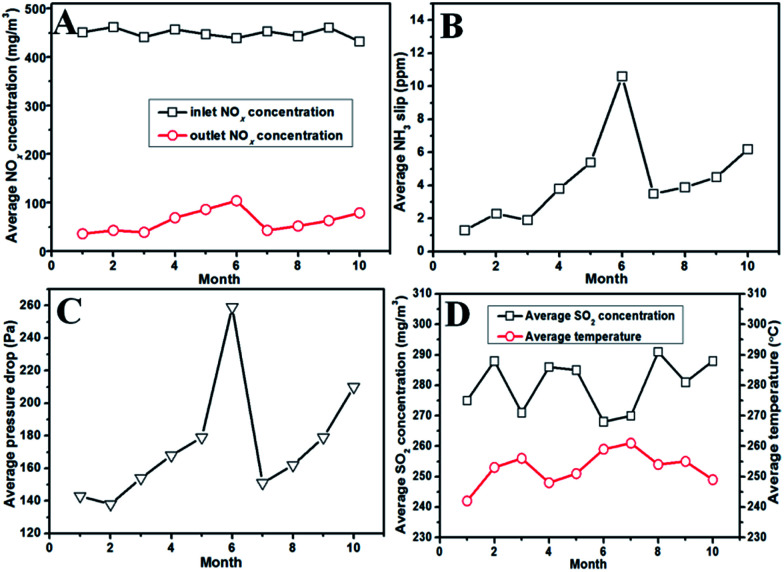
The results of the DeNO_*x*_ project case for ten months: (A) the average inlet/outlet NO_*x*_ concentration for each month, (B) the average NH_3_ slip for each month, (C) the average pressure drop through the SCR reactor for each month, and (D) the average outlet SO_2_ concentration and temperature for each month. The reactors were opened after having run for six months, and were reused after replacing the blocked foam metal plate and dredging the blocked channels of the honeycomb catalysts.

Fig. 2 shows the operating data for the DeNO_*x*_ project case in the past ten months. The conditions of the flue gas were relatively stable during the ten months. The average NO_*x*_ concentration was about 450 mg m^−3^, the average SO_2_ concentration was from 260–300 mg m^−3^, and the average temperature was from 240–260 °C. In the first three months, the NO_*x*_ concentration in the exit of the SCR reactors was below 50 mg m^−3^ with about 90% DeNO_*x*_ efficiency. Meanwhile, the NH_3_ slip and pressure drop were also stable at low levels. However, values for these data (the outlet NO_*x*_ concentration, NH_3_ slip and pressure drop) began to increase from the fourth month. The NO_*x*_ concentration for the sixth month was close to 150 mg m^−3^ together with a NH_3_ slip of more than 10 ppm, and even the pressure drop increased rapidly. The high pressure drop with an decreased DeNO_*x*_ efficiency implied that the honeycomb catalysts in SCR the reactors were probably blocked up. On the other hand, an attempt to clean the catalytic bed using the soot blower could not decrease the pressure drop. Thus, the spare reactors were used temporarily, and the used reactors were opened after having run for six months. Indeed, it was found that both the foam metal plates and honeycomb catalysts were severely blocked up by ash (the details are introduced in the next part). After replacing the blocked foam metal plate and dredging the blocked channels of the honeycomb catalysts, function of the used reactors was recovered, as shown, from the seventh to the tenth month. In the seventh month, the DeNO_*x*_ efficiency recovered close to the initial value, and the values for NH_3_ slip and pressure drop were a little higher than their initial values. However, the outlet NO_*x*_ concentration, NH_3_ slip and pressure drop increased gradually in the next three months (until now), which might also have resulted from the blocking of the SCR reactors.

## 4. Characterization of the unloaded catalyst sample

**Fig. 3:**
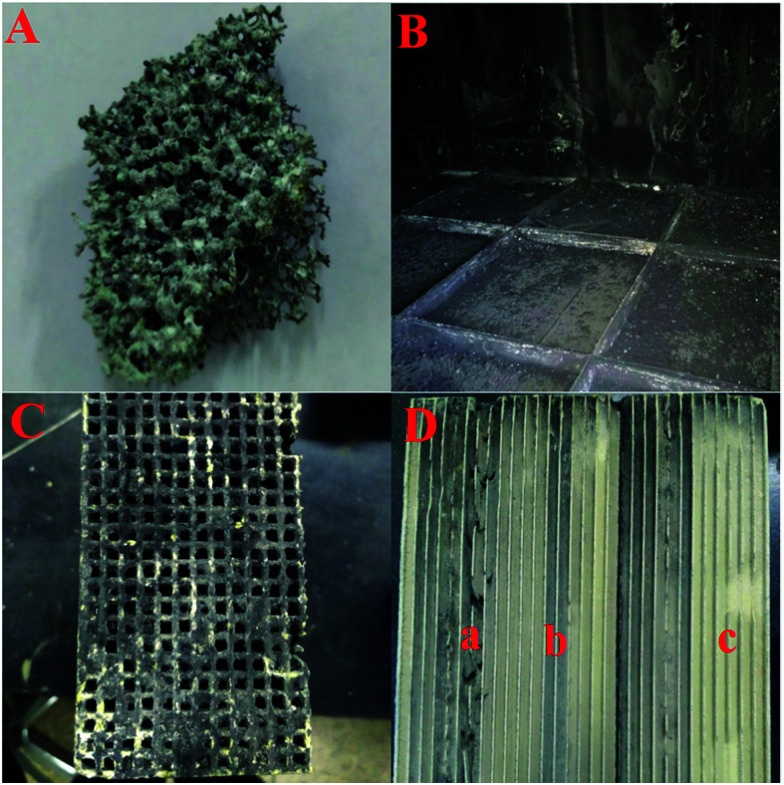
The digital photographs of the foam metal plate and catalyst after running for six months: (A) the foam metal plate, (B) the top of the honeycomb catalyst modules, (C) the side face of the honeycomb catalyst, and (D) the inner channels of the honeycomb catalyst with the three typical areas marked a, b and c.

To find out the reason for the decreased DeNO_*x*_ efficiency and increased pressure drop after the DeNO_*x*_ project case had been operating for six months, the used SCR reactors were opened and samples of both the foam metal plate and the honeycomb catalyst were also taken for further study in the lab. Fig. 3 shows the digital photographs of the used foam metal plate and catalyst. It can be seen that the foam metal plate was severely blocked by ash (Fig. 3A). Even worse, a thick layer of dust had been deposited and covered the upper side of the honeycomb catalyst modules (Fig. 3B). For a single honeycomb, it can be seen that about a quarter of the channels were blocked up (Fig. 3C). The coverage and blocking of the honeycomb channels by deposited ash would observably obstruct the normal flow of flue gas through the honeycomb catalysts, resulting in the increased pressure drop and decreased DeNO_*x*_ efficiency. However, the inner channels of the honeycomb catalyst were not blocked at all. Only some gray ash had been deposited on partial channels of the honeycomb. By close examination, interestingly, the ash was found to be deposited only on the channels with defects (derived from their manufacture, gray channel part “a” in Fig. 3D) or the channels with their upper side blocked up (gray channel part “b” in Fig. 3D). In contrast, nearly no ash was deposited on the channels without any blocking (yellow channel part “c” in Fig. 3D). These results indicated that the linear velocity of the flue gas through the honeycomb channels had a great impact on the deposition of ash on the inner surfaces of the honeycomb channels. Defective or blocked channels with low linear velocities facilitated the deposition of ash.

**Fig. 4:**
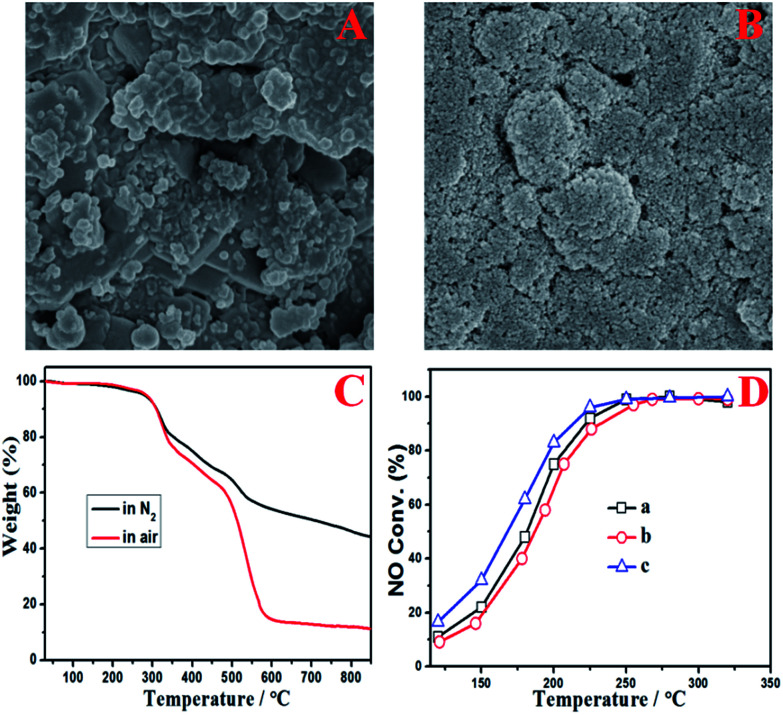
Electron microscopy images of the surface of the honeycomb channels: the gray channel part (A) and the yellow channel part (B); TG analysis in air or N_2_ of the gray ash from the catalyst surface (C); and activity testing of single-hole honeycomb (D): fresh catalyst (a), the gray channel part (b), and the yellow channel part (c).

More detailed characterization was carried out as shown in Fig. 4 to evaluate the influence of the deposited ash on the DeNO_*x*_ efficiency of catalyst itself. It can be seen from the gray channel that the catalyst particles were partially covered (Fig. 4A), but no covering was found on the surface of the yellow channel (Fig. 4B). Moreover, the thermogravimetric (TG) analysis of gray ash shown in Fig. 4C indicated that the ash contained about a 50 wt% carbon component (the weightlessness step from 480–580 °C in air) and about 40 wt% ammonium sulfate species (the weightlessness step from 250–480 °C in air or N_2_). The carbon component was from the fly ash from the coke oven, and the ammonium sulfate species were from the gas-phase reaction between SO_3_ and NH_3_ according to the previous results of our pilot test.^11^ No obvious weightlessness steps in air from 500–650 °C for iron sulfate species were observed, indicating that few iron sulfate species were produced after the pipeline underwent antiseptic treatment.^15^ Moreover, compared with the fresh catalyst, the yellow channel part of the used catalyst even had better catalytic DeNO_*x*_ activity owing to the sulfurization of V based catalysts.^16,17^ However, the activity only decreased slightly for the gray channel part of the used catalyst, which resulted from partial coverage of the catalytic surface by ash. Combined with the operating data in Fig. 2, it can be deduced that the main reason for the decreased DeNO_*x*_ efficiency and increased pressure drop was the blocking of the honeycomb channels and the foam metal plate but not obvious deactivation of the catalyst itself.

## 5. Problems and solutions

According to the above results, the ash on the catalyst was mainly composed of carbon and ammonium sulfate species. The carbon component was from the fly ash in the original flue gas. The ammonium sulfate species were the gas-phase reaction products of the injected NH_3_ and SO_3_, which were formed and then bonded with fly ash in the flue before the SCR reactors. After the flue gas entered the SCR reactors, the linear velocity of the flue gas decreased suddenly owing to the markedly larger inner diameter of the SCR reactors than the pipe diameter of the flue. The ash was then sedimentary and partly trapped by the foam metal plate. However, a lot of ash still dropped down and covered the upper side of the honeycomb catalyst modules, which blocked up the honeycomb channels. On the other hand, the inner channels of the honeycomb catalysts were relatively clear, and only a small amount of ash was mainly deposited on the defective or blocked channels. The activity of the catalyst itself could still be maintained even after having run for six months. Therefore, the decreased DeNO_*x*_ efficiency and increased pressure drop of the DeNO_*x*_ project case were caused by the blocking up of the honeycomb channels with deposited ash.

**Fig. 5:**
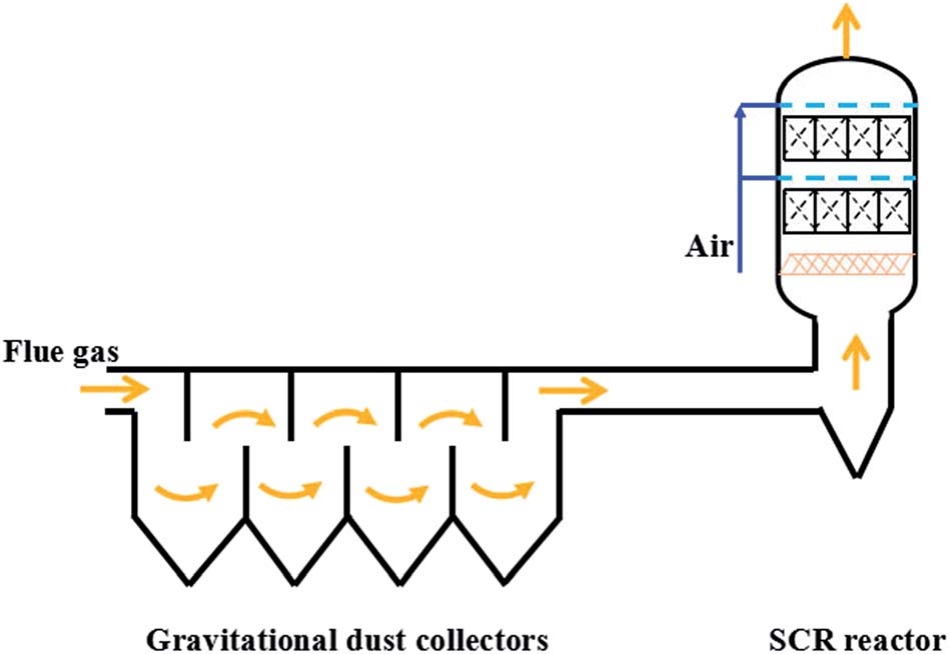
The possible improved DeNO_*x*_ process with gravitational dust collectors.

To solve these problems, an improved technological process was proposed, as shown in Fig. 5. In this process, gravitational dust collectors were added before where the flue gas was introduced into the SCR reactors, which were used to remove the ash of both fly ash and the ammonium sulfate species. Moreover, the flue gas flowed through the SCR reactor from the bottom to the top with an ash bucket in the entrance. The foam metal plate was placed below the honeycomb catalyst in the SCR reactors to trap the remaining ash and homogenize the flue gas. In this way, the deposition of ash on the catalyst would be alleviated to avoid the blocking up of the channels of the honeycomb catalyst. According to the operating data in Fig. 2, the lifetime of the catalyst would be more than one year if the problem of ash blocking was solved by the new process. On the other hand, the slow deactivation of the catalyst, which was caused by the covering on the catalytic surface as shown in Fig. 4, can also not be neglected. In addition to the carbon component, the covering also contained ammonium sulfate species. Therefore, the *in situ* regeneration craft was also necessary for the continuous and stable operation of the DeNO_*x*_ project case. The high-temperature gas from the hot-blast stove would burn out the covering to clear the catalyst surface and recover its intrinsic activity, in case the DeNO_*x*_ efficiency fell below the designed value. It is well known that a DeNO_*x*_ catalyst may be poisoned by ammonium sulfate species in the presence of SO_*x*_ at low temperatures.^18^ Although the poisoning by SO_*x*_ may be unavoidable in terms of the chemistry and the catalyst itself, the problem can be solved by advanced process engineering such as the elimination of SO_3_ using NH_3_, gravitational dust collection and *in situ* regeneration technology. The results of the project case in this work demonstrated that the engineering application of low temperature DeNO_*x*_ in the presence of SO_*x*_ is feasible in industry.

Moreover, the project case also has great cost advantages. In contrast, our other SCR unit was placed after the dry desulfurization and dedusting units for the purification of flue gas from steelworks, in which the flue gas temperature in the SCR reactors fell down from 260 °C to 200 °C. A much lower space velocity of 3000 h^−1^ as well as much higher content of active V component (5 wt%) had to be adopted for enough catalytic DeNO_*x*_ efficiency at 200 °C. The catalyst cost of the post-DeNO_*x*_ process per cubic flue gas was increased by about 40% compared with that of the pre-DeNO_*x*_ process reported in this work.

## 6. Conclusions

In summary, a DeNO_*x*_ project case was built and successfully run for the purification of low temperature flue gas in the presence of SO_*x*_ from a medium coking plant. New process engineering was considered and designed to overcome the problem of SO_*x*_, including the elimination of SO_3_ using NH_3_ before the SCR reactor, the filtration of ash using a foam metal plate and *in situ* regeneration technology. The project case could run continuously for six months with above 70% DeNO_*x*_ efficiency and less than 10 ppm for NH_3_ slip at 250 °C and a space velocity of 4000 h^−1^ in the presence of 260–300 mg m^−3^ SO_*x*_. The main problem of the project case was the blocking of the honeycomb channels by the sedimentary ash on the honeycomb catalyst modules, which resulted in decreased DeNO_*x*_ efficiency and an increased pressure drop. The ash was mainly composed of 50 wt% carbon component from the inherent fly ash and 40 wt% ammonium sulfate species from the gas-phase reaction between SO_3_ and NH_3_ in the flue after the injection of NH_3_. The sudden decrease in linear velocity from the pipeline to the SCR reactor resulted in severe deposition of ash on the upper side of the honeycomb catalyst modules, and the ash could also be deposited on the defective or blocked channel surfaces with a low linear velocity. Compared with the adverse effect of the ash, the effect of SO_2_ on the catalyst was not prominent for the low temperature SCR process, and the activity loss of the catalyst itself was not obvious even after having run for six months. A possible improved DeNO_*x*_ process was proposed with gravitational dust collectors to solve the ash blocking problems of the present project case, and the proposed process with the assistance of *in situ* regeneration technology is expected to realize continuous and stable operation of the industrial DeNO_*x*_ project for low temperature flue gas with high-concentration SO_*x*_.

## Conflicts of interest

There are no conflicts to declare.

## Supplementary Material
